# Cats and kids: how a feline disease may help us unravel COVID-19 associated paediatric hyperinflammatory syndrome

**DOI:** 10.1007/s15010-020-01515-3

**Published:** 2020-09-02

**Authors:** Martin Alberer, Ulrich von Both

**Affiliations:** 1 Division of Infectious Diseases and Tropical Medicine, University Hospital, Ludwig-Maximilians-University (LMU), Leopoldstrasse 5, 80802 Munich, Germany; 2 Division of Paediatric Infectious Diseases, Dr. von Hauner Children’s Hospital, University Hospital, Ludwig-Maximilians-University (LMU), Lindwurmstraße 4, 80337 Munich, Germany

To the editor,

With great interest we have been following latest news and reports on clusters of paediatric patients displaying clinical features of hyperinflammatory shock and a possible association with a SARS-CoV-2 infection [1]. Although most children show only a mild and uncomplicated course of COVID-19, in a small subset of paediatric patients severe symptoms including hyperinflammatory state, persistent fever, circulatory shock, and evidence of organ dysfunction have been reported. This novel syndrome is still a puzzle for clinicians and scientists since its underlying pathology is poorly understood making targeted treatment and preventive measures difficult. Similarities to children presenting with Kawasaki disease (KD) have been reported in some of these critically ill children while some of them predominantly display features of toxic shock, such as seen in severe staphylococcal or streptococcal infection. The RCPCH and CDC have published a case definition and scientists refer to this novel but still very rare severe clinical condition in children as “paediatric inflammatory multisystem syndrome temporally associated with SARS-CoV-2” (PIMS-TS).

While reflecting on this syndrome and its characteristic features, some interesting similarities come to mind when comparing the clinical course of PIMS-TS cases and the specific features of a disease in cats called feline infectious peritonitis (FIP) caused by the feline coronavirus (FCoV), an alphacoronavirus [2]. Both diseases show a predominance for the young. Cats are predominantly affected between 4 and 16 months of age which would relate to children and young persons in humans. Initially, a seemingly harmless viral gastrointestinal infection of the cat turns into a life-threatening systemic infection. Of particular note in this context, in two case series gastrointestinal symptoms have been the predominant feature of early PIMS-TS disease in almost all children [1, 3]. In cats, enterocytes are initially infected causing mostly mild gastrointestinal disease; but in a subset of infected cats (about 5%) a severe systemic infection arises after a variable period of time ranging from 2 weeks up to several months. In FIP, fibrinous and granulomatous serositis, protein-rich serous effusion in body cavities and/or granulomatous lesions develop [2]. Most of the children in the PIMS-TS case series of Riphagen et al. also showed ascites and pleural effusions [1]. Histopathologically, a granulomatous vasculitis is seen in FIP which relates to features of the Kawasaki syndrome although only small and medium sized vessels are primarily affected [2]. Furthermore, FIP shares additional features with severe COVID-19. Like in the human host, overexpression of inflammatory cytokines has been shown in FIP, particularly for TNF-α, IL-1β and IL-6 [2, 4]. Infected and subsequently activated monocytes/macrophages have been proposed to play a key role in this fulminant pathology [2]. In human COVID-19, these cytokines have been associated with a severe course of disease and deemed to be the hallmark cytokines of the cytokine storm. Infection of monocytes/macrophages with SARS-CoV-2 has been described, but it is still unclear whether these cells allow a permissive infection of the virus [5]. On the other hand, even non-permissive infection of macrophages and dendritic cells by SARS-CoV has been associated with significant overexpression of pro-inflammatory cytokines [6]. In addition, similar findings in white blood cell count, such as lymphopenia possibly caused by TNF-α- mediated apoptosis, are equally detected in both conditions and have been described in many human COVID-19 cases as well as in FIP [2].

The underlying pathophysiological mechanisms of FIP are still unclear. An in vivo mutation of FCoV affecting the viral spike protein (S-protein) and the occurrence of different pathogenic strains have been proposed in this context. On this note, it would be of great interest to see whether mutations in the viral genome, particularly in regions affecting the S-protein of SARS-CoV-2, could lead to a change in cell tropism enabling the virus to more effectively infect and replicate within human monocytes/macrophages subsequently leading to the clinical picture of PIMS-TS. Unfortunately, results on the pathological and histopathological examinations on patients with PIMS-TS, i.e. post-mortem pathology, are still missing. Closing this knowledge gap could help to clarify the underlying mechanisms of this challenging condition. Finally, RNA expression profiling of these cases in comparison to milder ones, like currently underway in the DIAMONDS study (https://www.diamonds2020.eu), will certainly add critical information on this aspect in the near future. Despite the fact that SARS-CoV-2 belongs to the genus of betacoronavirus and FCoV to the genus of alphacoronavirus the common link for both disease manifestations is the occurrence of multi-system vasculitis involving monocytes and macrophages as possible key players in the pathogenesis. With regards to other coronavirus infections, only for ferret systemic coronavirus (FRSC) a similar disease entity has been reported which closely resembles the non-effusive (dry) form of FIP. Interestingly, also in SARS-CoV infection a vasculitis with infiltration of monocytes and involvement of small veins has been described, a characteristic feature of FIP [7]. Other human and animal coronaviruses usually cause gastrointestinal (e.g. canine coronavirus, transmissible gastroenteritis virus) and/or respiratory disease (e.g. canine respiratory coronavirus, human coronavirus OC43) varying in individual degree of severity [8]. Infection of cats with SARS-CoV-2 is possible but up to now no infected animals showing symptoms and signs resembling FIP or PIMS-TS have been reported.

Even though currently known receptors for both viruses, ACE-2 and aminopeptidase N, are different, it has not been described that binding to these receptors triggers a receptor-specific effect besides mediating cell entry. Furthermore, the receptor for the type I of FCoV is not known yet. Although exact pathophysiological pathways of both viral infections are unfortunately still unclear, both viruses seem to have the potential to manipulate and evade the innate immune response by means such as the production of accessory proteins (e.g. accessory proteins 7a, 3a, 3b and non-structural protein 5 (nsp5) in the case of FCoV) or by interfering with the antiviral type I interferon response resulting in a hampered or delayed activation of this process [9, 10]. In the case of nsp5, this effect is mediated via negatively affecting the RIG-I-like receptor pathway which has also been shown for SARS-CoV and is also likely to play a role in SARS-CoV-2 infection [10, 11].

In our opinion, both viruses essentially manipulate the infected monocyte/macrophage leading to enhanced cytokine production. This results in an optimized environment for viral replication due to induced T-cell apoptosis and upregulation of both ACE-2 and aminopeptidase N, respectively. From a monocytic perspective, the cell is essentially locked in cytokine overdrive. Interestingly, remdesivir, currently the only drug with proven benefit on the clinical course of COVID-19, has also been successfully been used in the treatment of FIP.

We propose that FIP could be a promising veterinary disease to learn more about SARS-CoV-2 activity in monocytes/macrophages and help to elucidate the mechanisms underlying the profound production of cytokines resulting in a severe cytokine storm and PIMS-TS.
